# Use of pleural cryobiopsy for tissue culture specimens: a case report

**DOI:** 10.1002/rcr2.593

**Published:** 2020-06-05

**Authors:** Satoru Ishii, Manabu Suzuki, Hirokazu Arai, Jin Takasaki, Masayuki Hojo, Haruhito Sugiyama

**Affiliations:** ^1^ Department of Respiratory Medicine National Center for Global Health and Medicine Tokyo Japan

**Keywords:** Cryobiopsy, pleural disease, tissue culture, tuberculous pleurisy

## Abstract

A 43‐year‐old man presented with cough. His chest X‐ray showed a left‐sided pleural effusion. We suspected tuberculous pleurisy (TP), and thoracoscopy under local anaesthesia was performed. It showed entire pleura with scattered nodules. Nodules were biopsied by conventional biopsy forceps, but the tissue sample was small. Therefore, the nodules were biopsied with a cryoprobe. The tissue size obtained was 2 mm by conventional biopsy forceps, and 6 mm at 5 sec by cryobiopsy. Histological analysis of the conventional biopsy forceps and cryobiopsy specimen showed inflammation with lymphocytes and caseating granulomas. Tissue culture of conventional biopsy forceps was positive for *Mycobacterium tuberculosis*, and all sensitivity tests were positive. But, the tissue culture of the cryobiopsy sample was negative. There is a possibility that cryobiopsy is not useful for tissue culture for TP.

## Graphical abstract

We present the case of a 43‐year‐old man with suspected tuberculous pleurisy (TP). Thoracoscopy under local anaesthesia showed the entire pleura with scattered nodules. Nodules were biopsied by conventional biopsy forceps, but the tissue sample was small, so a cryobiopsy was performed. Histological analysis of the conventional biopsy forceps and cryobiopsy specimen showed inflammation with lymphocytes and caseating granulomas. Tissue culture of conventional biopsy forceps was positive for *Mycobacterium tuberculosis*, and all sensitivity tests were positive. But, the tissue culture of the cryobiopsy sample was negative. There is a possibility that cryobiopsy is not useful for tissue culture for TP.

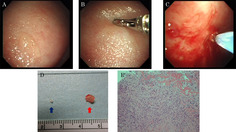

## Introduction

Thoracoscopy with local anaesthesia permits parietal pleural biopsies to be taken under direct vision. It can be performed without the need for intubation or single‐lung ventilation. It is a minimally invasive procedure with a low complication rate and low cost. It has also been reported as the most accurate tool for establishing not only carcinomatous pleurisy, but also tuberculous pleurisy (TP). It has recently been reported that cryobiopsy was useful for the diagnosis. Cryobiopsy was useful to obtain sufficient tissue, increase depth, and fewer instances of crush artefact than forceps biopsy [1]. But, it is not reported that tissue culture of cryobiopsy is effective.

## Case Report

**Figure 1:**
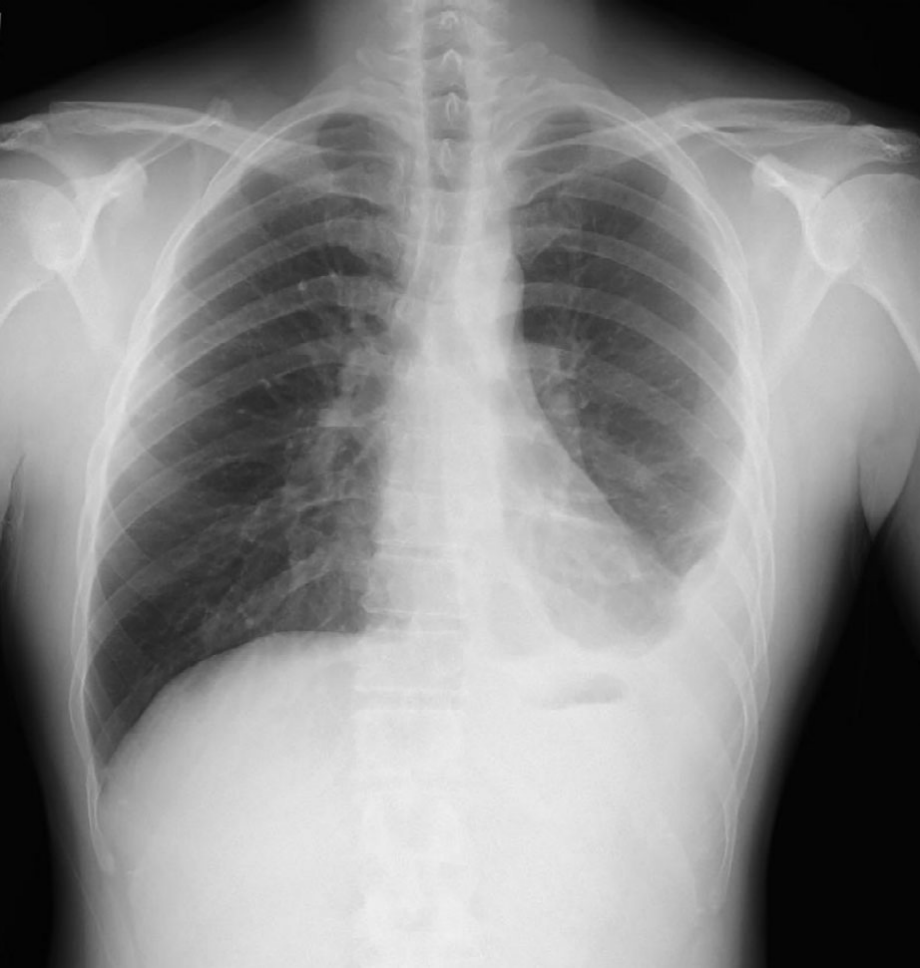
Chest X‐ray shows a left‐sided pleural effusion.

**Figure 2:**
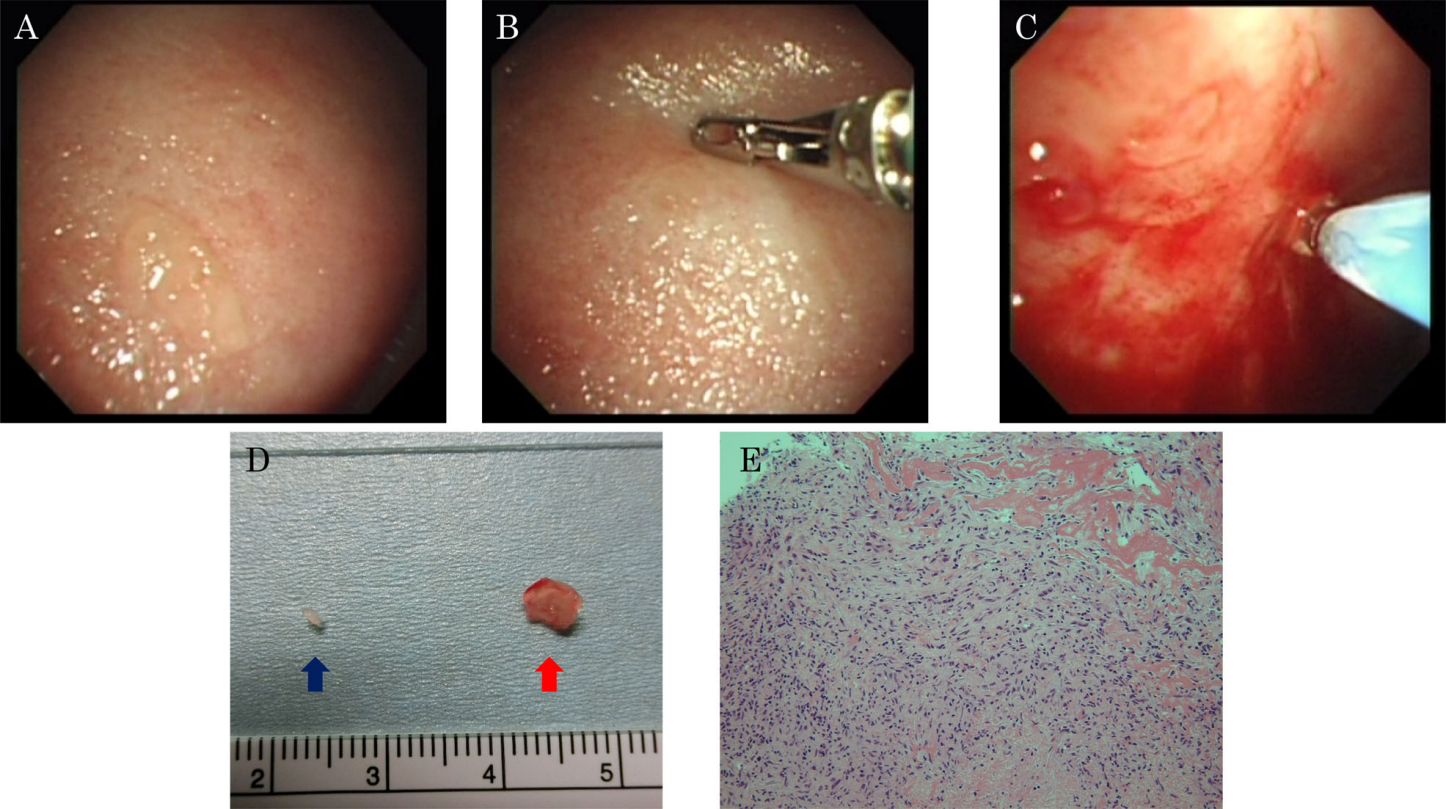
Thoracoscopy demonstrates entire pleura with scattered nodules (A). Conventional biopsy forceps biopsied the nodule (B). The tip of the cryoprobe was attached to the nodule and cooled using carbon dioxide (C). A tissue by conventional biopsy forceps (blue arrow) and a tissue at 5 sec (red arrow) by cryobiopsy (D). Histological analysis of the specimen showed inflammation with lymphocytes and caseating granulomas partially (E).

A 43‐year‐old man presented with cough. His chest X‐ray showed a left‐sided pleural effusion (Fig. 1). Thoracentesis indicated that the effusion was exudative, with adenosine deaminase of 84 U/L, and mycobacterial culture of pleural fluid was negative. Pleural cytodiagnosis was negative. We suspected TP or other diseases. Hence, thoracoscopy under local anaesthesia was performed using the LTF‐240 (Olympus, Japan). Thoracoscopy showed entire pleura with scattered nodules (Fig. 2A). Nodules were biopsied by conventional biopsy forceps (FB‐231D; Olympus) (Fig. 2B), but the tissue obtained was small. Therefore, the nodules were biopsied with a cryoprobe (2.0 mm probe; Erbe Elektromedizin, GmbH, Germany) at the same place. The tip of the probe was attached to nodules, and it was cooled once with carbon dioxide for 5 sec at the same place (Fig. 2C. The frozen tissue sample was extracted by pulling and released from the probe by thawing with normal saline. Slight bleeding was seen at the biopsied site, but it was stanched spontaneously. The tissue size obtained was 2 mm by conventional biopsy forceps, and 6 mm at 5 sec by cryobiopsy (Fig. 2D. Histological analysis of the conventional biopsy forceps and cryoprobe specimen showed inflammation with lymphocytes and caseating granulomas partially(Fig. 2E). Tissue culture of conventional biopsy forceps was positive for *Mycobacterium tuberculosis*, and all sensitivity tests were positive. But, the tissue culture of cryobiopsy was negative.

## Discussion

Pleural biopsy can be done via closed pleural biopsy or thoracoscopy under local anaesthesia. Closed pleural biopsy is reported to have high diagnostic rate for TP. But, closed pleural biopsy is a blind biopsy, and thoracoscopy under local anaesthesia permits parietal pleural biopsies to be taken under direct vision. Thoracoscopy establishes the diagnosis at a rate of nearly 100% for TP [2]. We performed thoracoscopy in this case. Pleural cryobiopsy is safe and consistently yields larger tissue specimens than flexible forceps biopsy [3, 4]. Shafiq et al. reported their review of 311 cryobiopsy procedures, and major bleeding was not reported in even one case [5]. Many reports were done that showed that cryobiopsy was useful to diagnose undiagnosed pleural effusion. To the best of our knowledge, few studies have reported that cryobiopsy is not useful for tissue culture for TP. TP was diagnosed by tissue samples or tissue cultures. Tissue sample had to demonstrate the presence of caseating granulomas. Tissue cultures had to be positive for *M. tuberculosis*. It was necessary not only to diagnose, but also to determine whether resistant tuberculosis is present. Treatment differs depending on whether resistance is found. Normally, treatment comprises four medications: isoniazid (INH), rifampicin (RFP), ethambutol (EB), and pyrazinamide (PZA), but if INH resistance is present, then RFP, PZA, EB, and streptomycin are mainly used, with the addition of levofloxacin and ethionamide when bacterial discharge is high [6]. The rate of positive cultures from pleural effusions in TP was low while the rate of positive cultures from tissues was high [5]. We biopsied the tissue, and some was used for pathology and some for tissue cultures. A tuberculosis bacillus is aerobe, its optimum growing temperature is 37°C, and optimum pH is 6.4–7.0. If the temperature is low, a tuberculosis bacillus will perish and the tissue culture will not grow. The cryoprobe was frozen with carbon dioxide for 5 sec to about −80°C. Tissue cultures obtained by cryobiopsy did not grow any culture while the tissue culture of conventional biopsy forceps was positive for *M. tuberculosis*, and all sensitivity tests were positive. But, the tissue culture of cryoprobe was negative in this case. There is a possibility that cryobiopsy is not useful for tissue culture for TP. It is necessary to collect many cases and this needs to be taken into consideration for the future.

### Disclosure Statement

Appropriate written informed consent was obtained for publication of this case report and accompanying images.
